# Differential mRNA and lncRNA Expression Profiles Associated with Early Pregnancy Loss in ART Patients

**DOI:** 10.1007/s43032-024-01576-x

**Published:** 2024-05-21

**Authors:** Liyan Wang, Yanbiao Jiang, Xiaorong Luo, Haofei Shen, Liulin Yu, Xia Yang, Hui Wang, Panpan Jin, Xuehong Zhang

**Affiliations:** 1 The First Hospital of Lanzhou University, Lanzhou, China; 2 The First School of Clinical Medicine, Lanzhou University, Lanzhou, China; 3 Key Laboratory for Reproductive Medicine and Embryo, Lanzhou, Gansu Province China

**Keywords:** Early pregnancy loss, RNA-seq, Chorionic villi tissues, Expression pattern

## Abstract

Early pregnancy loss (EPL) is the most common complication in assisted reproductive technology (ART). However, the precise causes for nearly 50% patients remain unexplained. In the current study, we aimed to discover the differentially expressed profiling of mRNAs and lncRNAs by RNA sequencing (RNA-seq). Human chorionic villi tissues were collected from patients with EPL and natural control (NC) group. RNA sequencing (RNA-seq) of these specimens was performed for transcriptome analysis. As a result, we identified a total of 141 mRNAs and 137 lncRNAs that were significantly differentially expressed between villi tissues from EPL and NC. Functional enrichment analyses indicated enrichment of differentially expressed genes involved in pathways were associated with growth hormone receptor binding, PI3K-Akt signaling pathway, Jak-STAT signaling pathway, transcriptional misregulation in cancer, metabolic pathways and Rap1 signaling pathway. Additionally, the co-expression networks (lncRNA–miRNA–mRNA) was constructed based on the correlation analysis between the differentially expressed RNAs.7 mRNAs and 6 lncRNAs were successfully technically validated with RT-PCR. In conclusion, our results suggest a direction for the further study of EPL-related mRNAs and lncRNAs and may ultimately assist in understanding the pathogenesis of EPL.

**Supplementary Information:**

The online version contains supplementary material available at 10.1007/s43032-024-01576-x.

## Introduction

Early pregnancy loss (EPL) is the most common complication in early pregnancy, affecting 15.3% of clinically recognized pregnancies [1].More than 90% of pregnancy losses occur in the first trimester of pregnancy [2],which is referred to as EPL. Furthermore, patients undergoing assisted reproductive technology (ART) have a high EPL ratio of 14.7% [3]. EPL is usually associated with embryonic chromosomal [4], diabetes [5], endocrine disorders [6], reproductive immune [7], infection [8], and maternal–fetal interface [9]. However, the precise causes for nearly 50% patients remain unexplained (due to unknown etiology) and few treatment options existed for women with EPL. Moreover, the occurrence of EPL will result in poor results, such as serious physical and psychological trauma and significant economic losses, specifically in the setting of extremely desired pregnancy achieved via ART. Thus, it is necessary to uncover molecular events involved in EPL and achieve an improved understanding of its potential pathogenesis.

In the human genome, protein-coding genes account for only around 2%, constitute less than 2% of the whole genome sequence, while the remained 98% is predominantly recognized as non-coding RNA (ncRNA). Long non-coding RNAs (lncRNAs), which are a subclass of the most studied non-coding RNA types, are more than 200 nucleotides in length. They play vital roles in multiple aspects of the biological processes, including epigenetic changes, transcriptional, cell cycle regulation, miRNA sponge, post-transcriptional regulation and participation in signaling pathways [10]. While the roles of lncRNA-mRNA interaction networks in EPL and their underlying mechanisms have been continuously investigated, related data of research articles remain scarce. Next-generation sequencing provides a high-throughput method for exploring the diverse, poly-adenylated RNA populations. This method can provide accurate identification and quantitation of messager RNAs (mRNAs) and other ncRNAs, such as lncRNAs.

Therefore, in the present study, we performed an RNA-seq analysis of chorionic villi tissues from patients with EPL and control group to analyze the differentially expressed profiling of lncRNAs and mRNAs. Furthermore, we validated several differentially expressed mRNAs and lncRNAs using quantitative real-time RT–PCR (qRT–PCR). Finally, we constructed a CeRNA network in EPL according to the predicted miRNA–mRNA and lncRNA–miRNA pairs.

## Materials and Methods

### Patients and Samples

Between June 2020 and December 2021, a total of 20 EPL patients and 18 NC women were recruited at the First Hospital of Lanzhou University. Termination of pregnancy in both group occurred at 7–12 weeks’ gestation. The pregnancies in natural control (NC) group were voluntarily terminated for non-medical reasons. EPL was defined as a spontaneous intrauterine pregnancy loss before the 12th gestational week. Patients with abnormalities (such as uterine malformations, untreated septate uterus, adenomyoma, submucous uterine fibroids, endometrial polyps or untreated intrauterine adhesions), hormonal abnormality, endocrine disease, karyotype abnormality, thyroid dysfunction, or infection were excluded. Human chorionic villi tissues were collected and stored at − 80 °C after saturating the RNAwait (Solarbio, Beijing, China).The basic situation comparison between the two groups of research objects and the treatment status of ART patients were respectively showned in Supplementary Table 1 and Supplementary Table 2.

### Total RNA Extraction

Total RNA was isolated from villi tissues using Trizol reagent (Qiagen, Hilden, Germany). RNA degradation and contamination were detected using 1% agarose gels*.* RNA concentration and purity were measured by NanoDrop ND 1000 spectrophotometer (NanoDrop Technologies, Wilmington, DE, USA), and total RNA integrity was assayed using the RNA Nano 6000 Assay Kit of the Bioanalyzer 2100 system (Agilent Technologies, CA, USA). Only RNA samples with high-quality (RNA integrity number ≥ 7.0) were used for further experiments.

### Library Preparation for mRNA/lncRNA and Sequencing

A total of 9 villi tissues (4 NC and 5 EPL) were sent to Novegene Biotechnology Co, Ltd (Beijing, China) for transcriptome sequencing. Briefly, the ribosomal RNA(rRNA) was removed from total RNA. Subsequently, sequencing libraries were established using NEBNext® UltraTM RNA Library Prep Kit for Illumina® (NEB, USA) according to the manufacturer’s instructions, and library quality was assessed on the Agilent Bioanalyzer 2100 system. The clustering of the index-coded samples was performed on a cBot Cluster Generation System using TruSeq PE Cluster Kit v3-cBot-HS (Illumia) according to the manufacturer’s instructions. After cluster generation, the library preparations were sequenced on an Illumina Hiseq platform and 125 bp/150 bp paired-end reads were generated.

### Filtering of Clean Reads

Raw data (raw reads) of fastq format were firstly processed through in-house perl scripts. In this step, clean data (clean reads) were obtained by removing reads containing adapter, reads containing ploy-N and low quality reads from raw data. At the same time, Q20, Q30 and GC content the clean data were calculated. All the downstream analyses were based on the clean data with high quality.

### Differential Expression Analysis

HTSeq v0.6.0 was used to count the reads numbers mapped to each gene. The expression levels for mRNAs and lncRNAs were determined by calculating the fragments per kilobase of exon model per million reads mapped (FPKM).The differential expression of mRNAs and lncRNAs was evaluated using the edgeR R package (3.12.1). The P values were adjusted using the Benjamini & Hochberg method. Padj < 0.05 and log_2_ FoldChange > 1 were set as the threshold for significantly differential expression. The volcano plots and heatmap were plotted by http://www.bioinformatics.com.cn, a free online platform for data analysis and visualization. orthogonal partial least-squares discriminant analysis (OPLS-DA) was generated using MetaboAnalyst 5.0 (http://www.metaboanalyst.ca).

### Enrichment Analysis of Function and Pathway

Gene Ontology (GO) enrichment analysis were carried out for differentially expressed mRNAs, adjacent genes of differentially expressed lncRNAs (adjacent gene refers to the gene closest to the lncRNA on the genome) using the R package GOseq. Kyoto Encyclopedia of Genes and Genomes (KEGG) enrichment analysis of differentially expressed genes was performed by KOBAS v3.0 software to obtain the enriched pathways of differentially expressed genes. Corrected *P*-value < 0.05 was considered statistically significant in GO terms and KEGG pathways.

### CeRNA Network Contruction

We used the miRcode database (http://www.mircode.org/) [11] to predict the targeted miRNAs of differentially expressed lncRNAs. Next, the miRDB (http://www.mirdb.org/) [12] andTargetScan (http://www.targetscan.org/) [13] databases were used to obtain the the differentially expressed mRNA-miRNA interaction pairs. To improve the effectiveness of our results, only the overlapping of the interaction pairs supported by the two databases was retained to create the miRNA-mRNA pairs. Finally, the lncRNA-miRNA-mRNA network was constructed and visualized by Cityscape (version3.8.1) software.

### Quantitative Reverse Transcription PCR (RT-qPCR)

A total of 29 villi tissues (14 NC and 15 EPL), not including those in RNA-seq analysis, were used to validate the differentially expressed RNAs by RT-qPCR. According to the manufacturer’s instructions, total RNAs were extracted from tissue samples by TRIzol reagent (Qiagen, Hilden, Germany). The RNA was purified and reverse transcribed to cDNA using PrimeScript RT Reagent Kit (Takara). RT-qPCR reactions were carried out using PerfectStart™ Green qPCR SuperMix (TransGen Biotech) on the ABI 7500 system (Applied Biosystems, Foster City, CA,USA) to detect the relative expression levels of mRNAs and lncRNAs. All samples were run in triplicate, and relative expression level was calculated using the 2^−^△△CT method relative to GAPDH. The RT-qPCR primer sequences are listed in Supplementary Table 3. Statistical analyses were carried out using GraphPad Prism software (version 9.0). *P* < 0.05 was considered statistically significant.

## Results

### Identification of Differentially Expressed mRNAs and lncRNAs

**Fig. 1:**
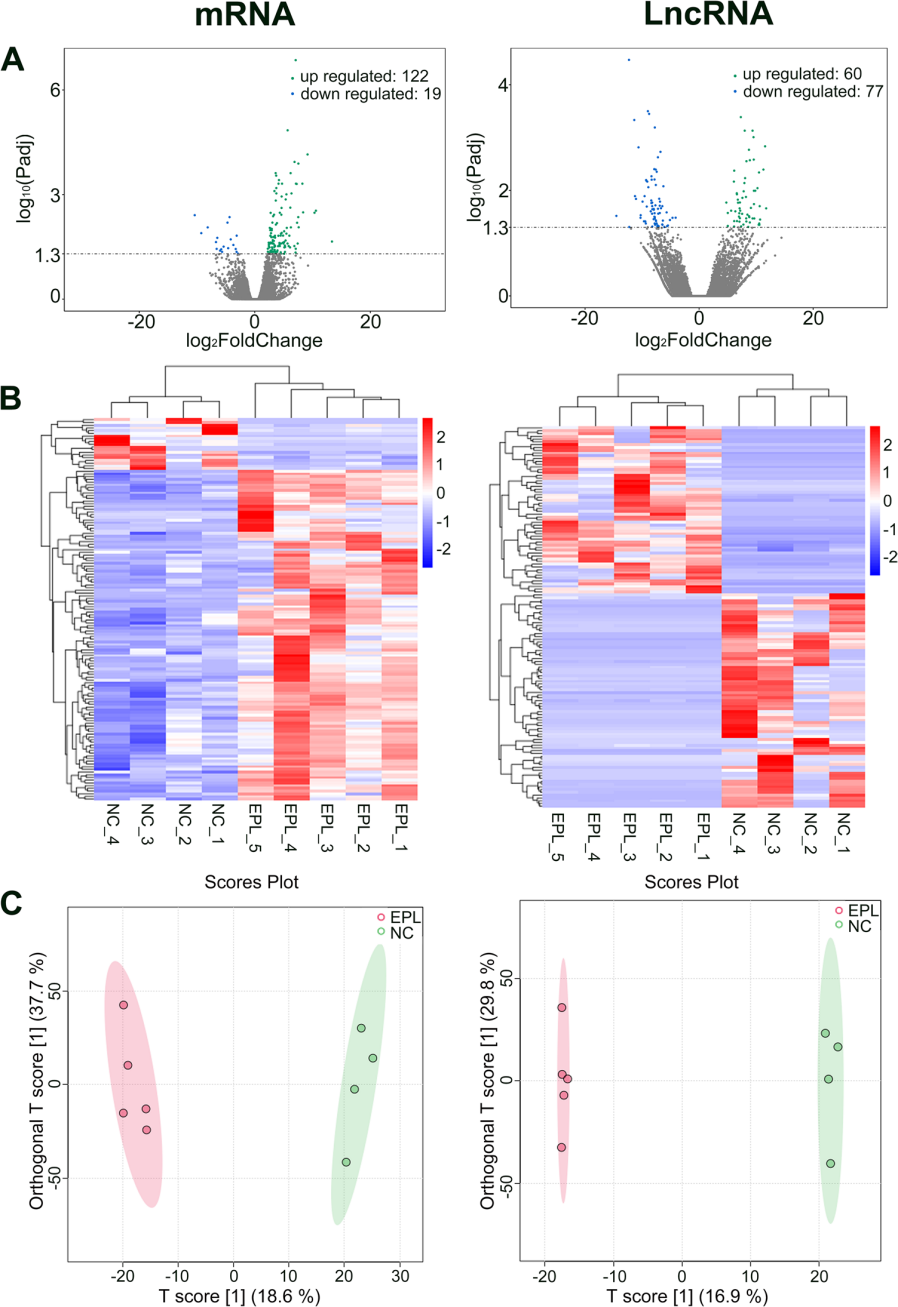
Analysis of differentially expressed mRNAs and lncRNAs in villi tissues from NC and EPL. **A** Volcano plots of differentially expressed mRNAs and lncRNAs. Green indicates high expression and blue represents low expression. **B** Heatmaps of differentially expressed mRNAs and lncRNAs. **C** *OPLS*-*DA* of NC(green) and EPL (pink) samples show distinct clustering

To investigate gene transcription changes of EPL patients, we performed a comparative transcriptomic analysis of the villi tissues from 5 EPL patients vs 4 NC women. In total, there were 141 differentially expressed mRNAs (122 upregulated and 19 downregulated) (Supplementary Table 4) and 137 differentially expressed lncRNAs (60 upregulated and 77 downregulated) (Supplementary Table 5) between NC and EPL patients. These differentially expressed mRNAs and lncRNAs are displayed using volcano plot (Fig. 1A),heat map (Fig. 1B) respectively. The orthogonal partial least-squares discriminant analysis (OPLS-DA) showed that the villi tissues from EPL patients exhibited distinct expression profiles compared with controls (Fig. 1C). The above results indicated that significant differences in mRNAs and lncRNAs expression patterns existed between NC and EPL.

### GO and Pathway Analyses of mRNAs

**Fig. 2:**
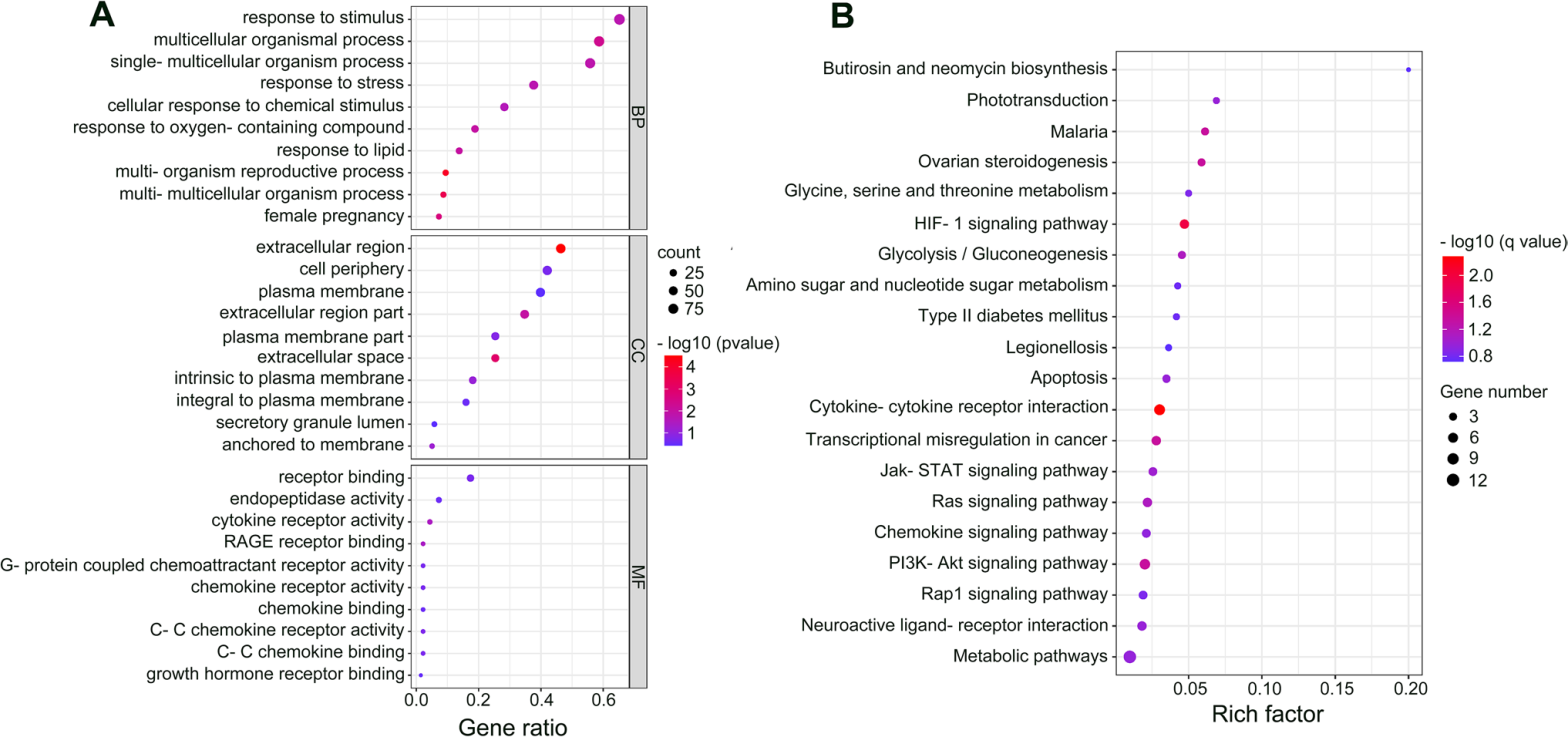
GO and KEGG pathway enrichment analyses of differentially expressed mRNAs. **A** The top 10 enriched GO terms for differentially expressed mRNAs. BP indicates biological process, CC indicates cellular component, and MF indicates molecular function. **B** The top 20 enriched KEGG pathways of all differentially expressed mRNAs

GO analysis demonstrated that the main terms were biological processes (BP) in which differentially expressed mRNAs were involved. The top ten most enriched GO terms in each category were presented in Fig. 1c, showing that the most enriched GO terms were response to stimulus, extracellular region and receptor binding in corresponding biological process category, cellular component category and molecular function category (Fig. 2A), respectively. Furthermore, KEGG pathway analyses displayed that these differentially expressed mRNAs were enriched in several pathways, including cytokine-cytokine receptor interaction, HIF-1 signaling pathway, PI3K-Akt signaling pathway, and transcriptional misregulation in cancer (Fig. 2B).

### LncRNA Function Annotation

**Fig. 3:**
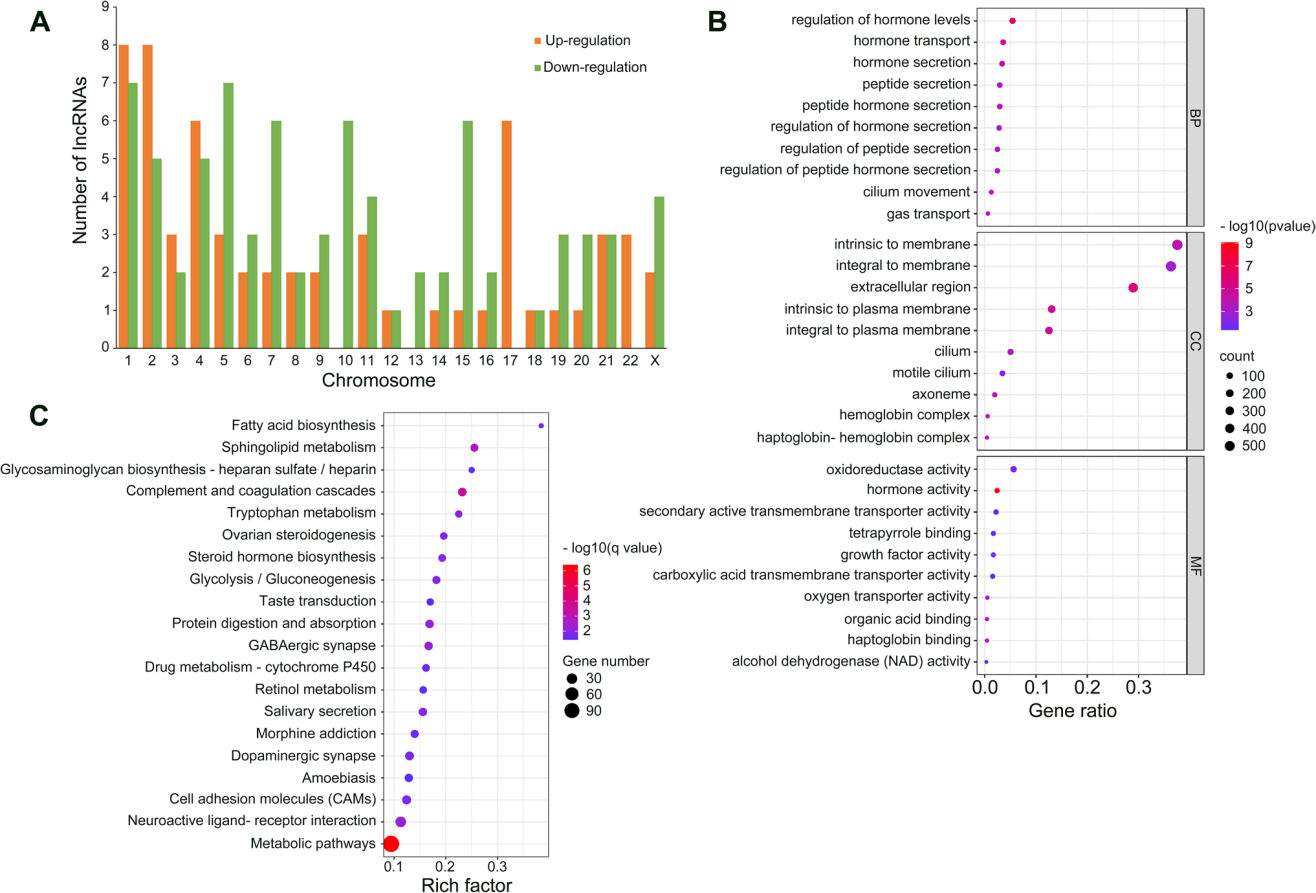
Expression profile of lncRNAs from NC and EPL. **A** The differentially expressed lncRNAs were classified according to their percentages in different chromosomes. Orange indicates high expression and green represents low expression. **B** The top 10 enriched GO terms for differentially expressed lncRNAs. BP indicates biological process, CC indicates cellular component, and MF indicates molecular function. **C** The top 20 enriched KEGG pathways of all differentially expressed lncRNAs

To further gain insights into the biological functions of dysregulated lncRNAs in EPL, a co-expression analysis was performed to predict target mRNAs. Chromosomal distribution indicated that the upregulated lncRNAs were located on all the chromosomes except chromosome 10 and 13, with more lncRNAs being located on chromosomes 1, 2. The downregulated lncRNAs were located on all the chromosomes with the exception of chromosome 17 and 22, which were predominantly located on chromosomes 1 and 5 (Fig. 3A). GO and KEGG pathway analyses were used for the target mRNAs of the lncRNAs. GO analysis of differentially expressed lncRNA target genes showed that the most enriched GO terms were gas transport in BP category, haptoglobin-hemoglobin complex in CC category, and alcohol dehydrogenase (NAD) activity in MF category (Fig. 3B). Additionally, KEGG pathway analysis indicated that the target genes of differentially expressed lncRNAs were enriched in several pathways, including metabolic pathways, complement and coagulation cascades, sphingolipid metabolism, and neuroactive ligand-receptor interaction (Fig. 3C).

### Validation of Differentially Expressed mRNAs and lncRNAs by RT-qPCR

**Fig. 4:**
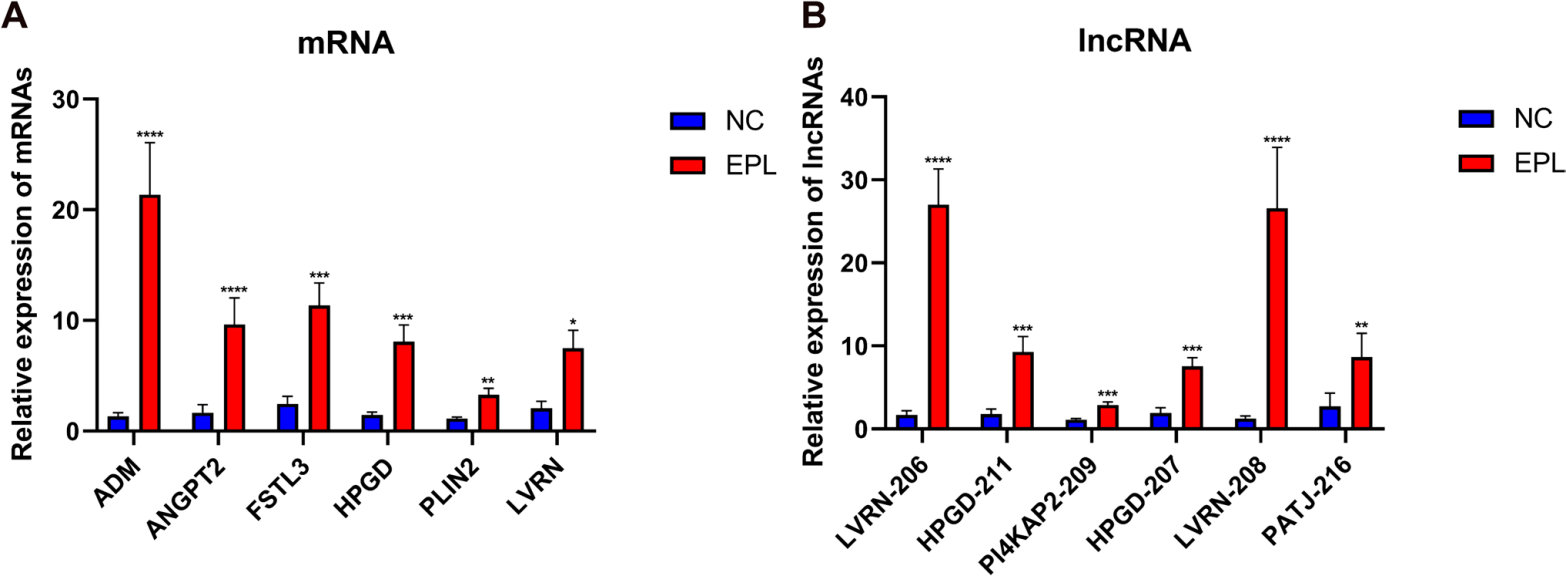
Validation of differentially expressed mRNAs and lncRNAs in NC and EPL group. (**A**) The expression levels of 6 mRNAs detected by RT-qPCR. (**B**) The expression levels of 6 lncRNAs detected by RT-qPCR. The data are presented as the mean ± SEM. *P < 0.05; **P < 0.005; ***P < 0.0005; ****P < 0.0001

To verify the accuracy of the RNA-seq data, we selected some differentially expressed mRNAs and lncRNAs and measured their expression levels by RT-qPCR in an independent set of NC (n = 14) and EPL (n = 15) villi tissues. The present results indicated that the mRNA expression levels of ENSG00000148926 (ADM), ENSG00000091879 (ANGPT2), ENSG00000070404 (FSTL3), ENSG00000164120 (HPGD), NSG00000147872 (PLIN2) and ENSG00000172901 (LVRN) were upregulated in EPL compared to NC (Fig. 4A). Moreover, comparing EPL group with NC, the lncRNA expression levels of ENST00000506279 (LVRN-206), ENST00000512410 (HPGD-211), NST00000479693 (PI4KAP2-209), ENST00000509512 (HPGD-207), ENST00000512413 (LVRN-208) and ENST00000635214 (PATJ-216) were upregulated (Fig. 4B). Therefore, the experiment showed results similar to the RNA-seq data.

### Construction of the CeRNA Regulatory Network

**Fig. 5:**
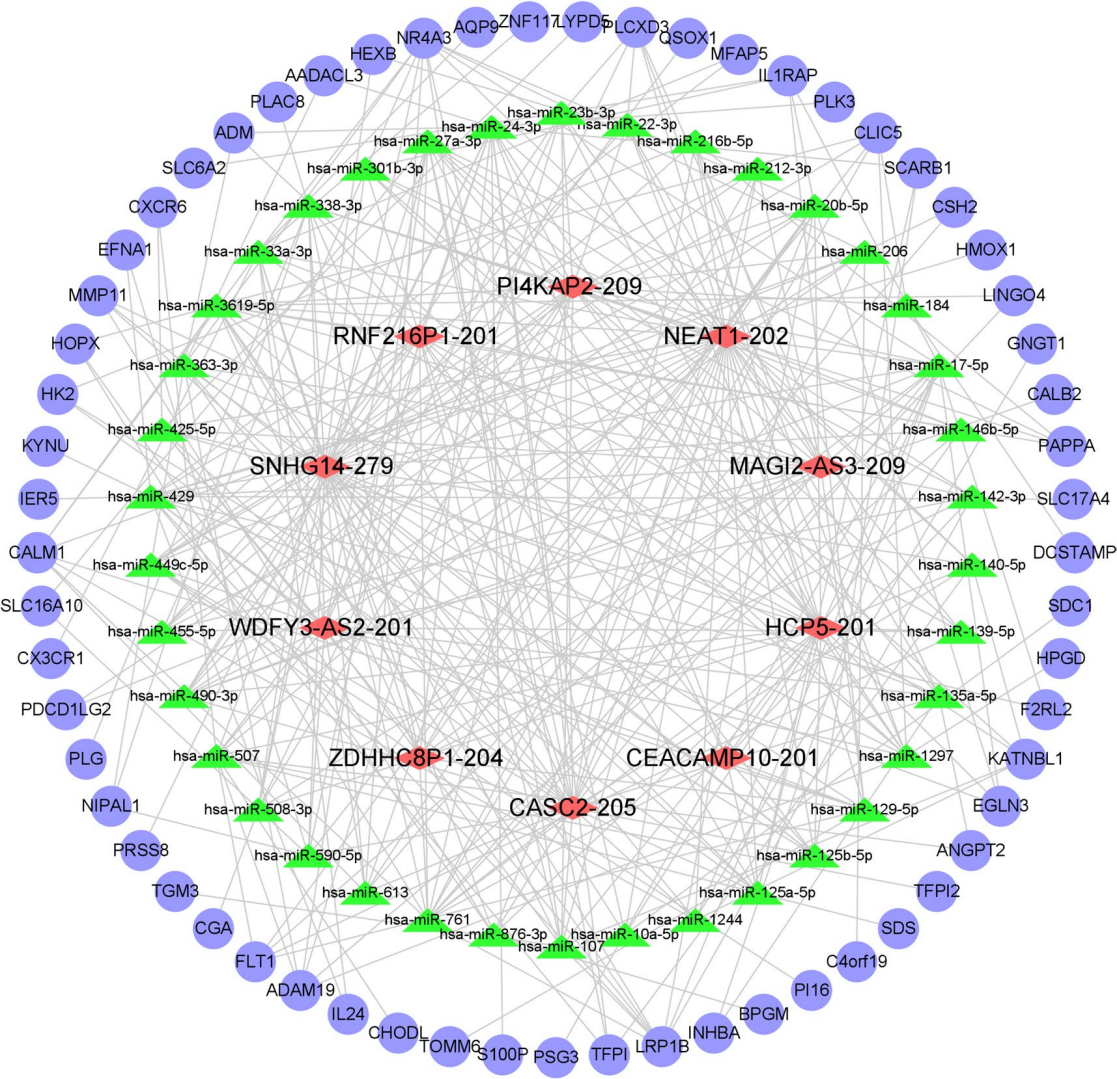
Integrated lncRNAs-miRNA-mRNA co-regulation CeRNA network analysis. Red diamond-shaped nodes represent lncRNA; green triangle indicates miRNA; blue circular nodes represent mRNA

We predicted the miRNAs interacting with differentially expressed lncRNAs or mRNAs using the miRNA target prediction software. The differentially expressed mRNAs and lncRNAs that shared the common miRNAs were incorporated into the construction of the CeRNA network. As shown in Fig. 5, the CeRNA network contained 10 lncRNAs, 38 miRNAs, and 61 mRNAs.

## Discussion

In the present study, we investigated the expression profile of mRNAs and lncRNAs in EPL patients using the RNA-seq technology. We found that a total of 141 mRNAs and 137 lncRNAs were differently expressed in EPL villi tissues compared to controls. Several differently expressed lncRNAs and mRNAs were validated by qRT-PCR. GO and KEGG pathway analysis showed that these RNAs largely involved in the growth hormone receptor binding, PI3K-Akt signaling pathway, Jak-STAT signaling pathway, Transcriptional misregulation in cancer, Metabolic pathways and Rap1 signaling pathway.

Upon RNA-seq, we identified 141 differentially expressed mRNAs between EPL and NC tissues, among which adrenomedullin(ADM), angiopoietin 2(ANGPT2), perilipin 2(PLIN2), follistatin like 3(FSTL3), hydroxyprostaglandin dehydrogenase(HPGD) and the low density lipoprotein receptor protein 1B(LRP1B) were further confirmed by real-time PCR.

ADM is a multifunctional peptide that supports vascular activity and placental growth,inhibitiing its function can lead to fetus-placental growth restriction and damage of placental blood vessels [14],. In addition, ADM can promote trophoblast invasion..A recent study [15] showed increased expression of ADM in trophoblast cell lines(HTR-8/SVneo) cultured under hypoxia, regulated by HIF-1-dependent pathway, resulting in enhanceed trophoblast invasion activity.The results of this study show that the mRNA expression level of ADM in EPL group is upregulated, indicating that the EPL patients may have insufficient angiogenesis and lack of oxygen, leading the compensatory rise of ADM.However,another study [16],showed that the serum ADM level of pregnant women on the day of embryo transfer was higher than that of non-pregnant women (P > 0.05), but the baseline ADM value is missing.The differential expression of ADM in serum and endometrium still needs further study..

ANGPT2 can stimulate the invasion and migration of trophoblastic cells in early pregnancy [17],participate in vascular remodeling and decidua angiogenesis, and is crucial for embryonic development [18]. Studies have shown that in mice, overexpression of ANGPT2 leads to failure of angiogenesis, resulting in embryo death [19]. In additional, abnormal ANGPT2 levels are associated with spontaneous abortion [20].In this study, ANGPTA2 levels increased in the EPL group, indicating that high levels of ANGPTA2 are adverse to pregnancy, which is consistent with these findings.

FSTL3 is also involved in the invasion, migration and proliferation of trophoblasts [21]. A recent study [22] on the relationship between FSTL3 and EPL showed that the level of FSTL3 in villus tissues of EPL group (n = 20) was significantly higher than that of normal pregnant women (n = 20) (*P* < 0.05), and the experimental results in mice showed that, the number of embryos implanted in the FSTL3 silenced group was significantly lower than that in the control group (*P* < 0.001), suggesting that FSTL3 plays a protective role in EPL women by neutralizing activin A.The results of this study are the same.

Previous studies have shown that 15-HPGD plays an important role in pregnancy maintenance, the expression of 15-HPGD in chorionic trophoblast cells of women with preterm delivery is lower [23]. A recent study [24] further demonstrated the indispensable role of 15-HPGD in the establishment of early pregnancy. The pregnancy rate of 15-HPGD knockout (15-HPGD-/-) matings was significantly lower than that of wild-type (15-HPGD + / +) matings(*P* < 0.01), and necrosis and inflammatory congestion were common at the implantation site of 15-HPGD-/- mice on the 8.5 day of gestation, and pregnancy loss occurred within 2 weeks of gestation.In this study, the upregulated expression of HPGD in EPL may be related to the increased compensation of beneficial factors in the early stage of embryo implantation. Due to the lack of research on the relationship between HPGD and EPL, it is necessary to further explore its mechanism of action, which also provides a new idea for the study of human early pregnancy loss.

Nevertheless, additional studies are necessary to explore the complex functions of these altered genes in EPL.

In the present study, KEGG pathway analysis showed that the differentially expressed mRNAs (Fig. 2B) and lncRNAs (Fig. 3C) were significantly enriched in PI3K-Akt, Jak-STAT, Ras and Rap1 signaling pathways. According to previous evidence, these pathways may affect the trophoblast function and further involved in pregnancy loss. For instance, PI3K/Akt signaling pathway significantly contributed to growth, migration and invasion of trophoblasts [25, 26], and the JAK/STAT pathway has also been demonstrated to associated with the growth of extravillous cytotrophoblast cells [27]. However, the precise mechanisms of these signaling pathways in EPL remain to be identified.

We are aware of several potential limitations in the current study. First, the number of samples for RNA-seq was limited. In order to get more accurate data, the characteristics of the subjects used for the RNA-seq, age, BMI, and gestational age were matched. Then, the qPCR validation results of 29 samples in our study were consistent with the result of RNA-seq. Second, placental villi tissue is comprised of several cell types, mainly trophoblast cells but also immune cells such as NK cells, macrophages, T cells, and monocytes. We choosed mixed sample for RNA-seq, which could require integrated studies of multiple cell types related with EPL. Third, we have not made in-depth functional studies. Future studies are needed to reveal the mechanisms by which the identified differentially expressed genes in EPL.Finally, the subjects of this study were patients with EPL during ART, considering the fact that almost no ART patients voluntarily terminated their pregnancy for non-medical reasons, patients with natural pregnancy were selected as the control group. Therefore, the differential expression of RNAs found in the EPL group may be related not only to the risk of EPL itself, but also to infertility, which is an unavoidable limitation of this study. In the future, we can also design a comparative study of non-medical causes and early abortion after natural pregnancy.

## Conclusion

In the present study, we investigated the expression profile of mRNAs and lncRNAs in EPL patients using the RNA-seq technology. We found that a total of 141 mRNAs and 137 lncRNAs were differently expressed in EPL villi tissues compared to controls. Several differently expressed mRNAs and lncRNAs were validated by qRT-PCR. GO and KEGG pathway analysis showed that these RNAs largely involved in the growth hormone receptor binding, PI3K-Akt signaling pathway, Jak-STAT signaling pathway, Transcriptional misregulation in cancer, Metabolic pathways and Rap1 signaling pathway. Our results suggest a direction for the further study of EPL-related mRNAs and lncRNAs and provide potential therapeutic targets for EPL.

## Supplementary Information

Below is the link to the electronic supplementary material.

Supplementary file1 (PNG 29 KB)

Supplementary file2 (PNG 172 KB)

Supplementary file3 (PNG 38.0 KB)

Supplementary file4 (PNG 760 KB)

Supplementary file5 (PNG 879 KB)

## Data Availability

Not applicable.
