# Supplementary figures and images for: Differential mRNA and lncRNA Expression Profiles Associated with Early Pregnancy Loss in ART Patients
Source: Reprod Sci. 2024 May 21;32(1):229–37. doi: 10.1007/s43032-024-01576-x (PMC11729186; doi:10.1007/s43032-024-01576-x)

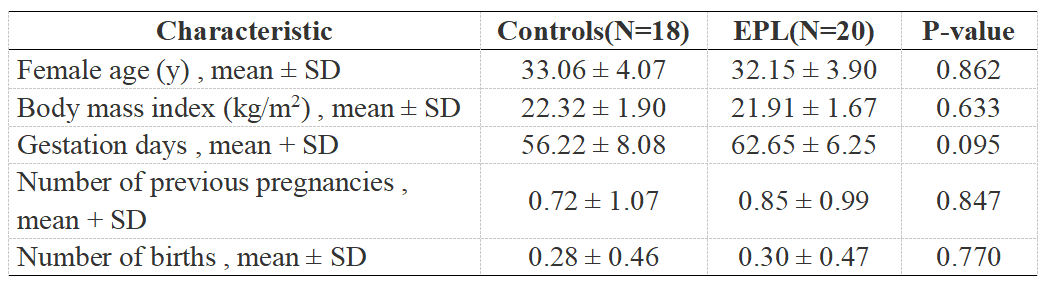

Supplement: Supplementary file 1 — Supplementary file1 (PNG 29 KB) [file 43032_2024_1576_MOESM1_ESM.png]

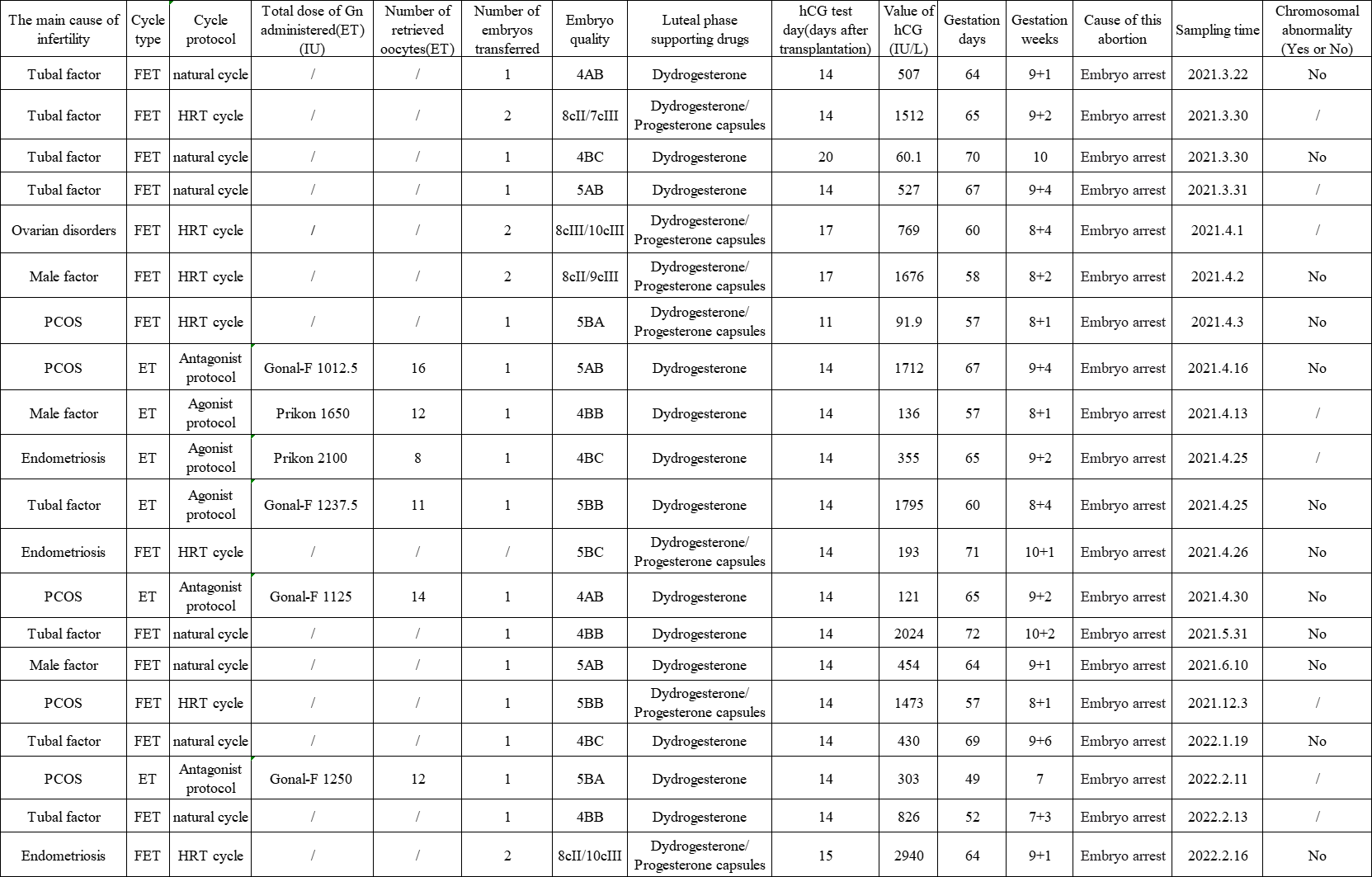

Supplement: Supplementary file 2 — Supplementary file2 (PNG 172 KB) [file 43032_2024_1576_MOESM2_ESM.png]

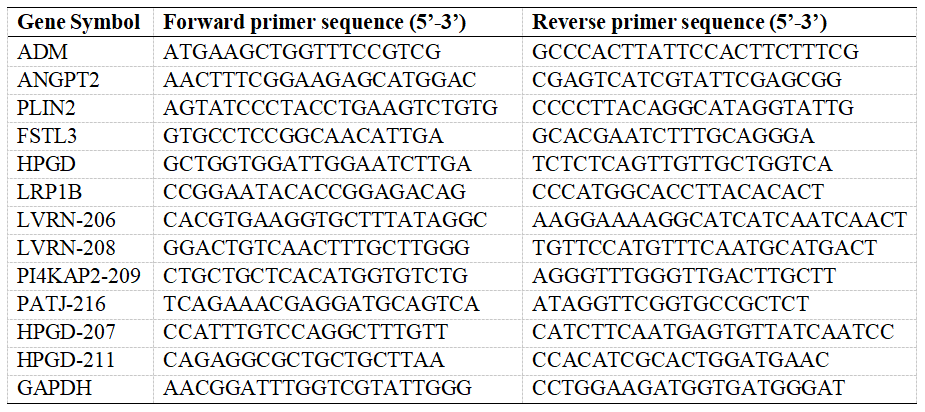

Supplement: Supplementary file 3 — Supplementary file3 (PNG 38.0 KB) [file 43032_2024_1576_MOESM3_ESM.png]

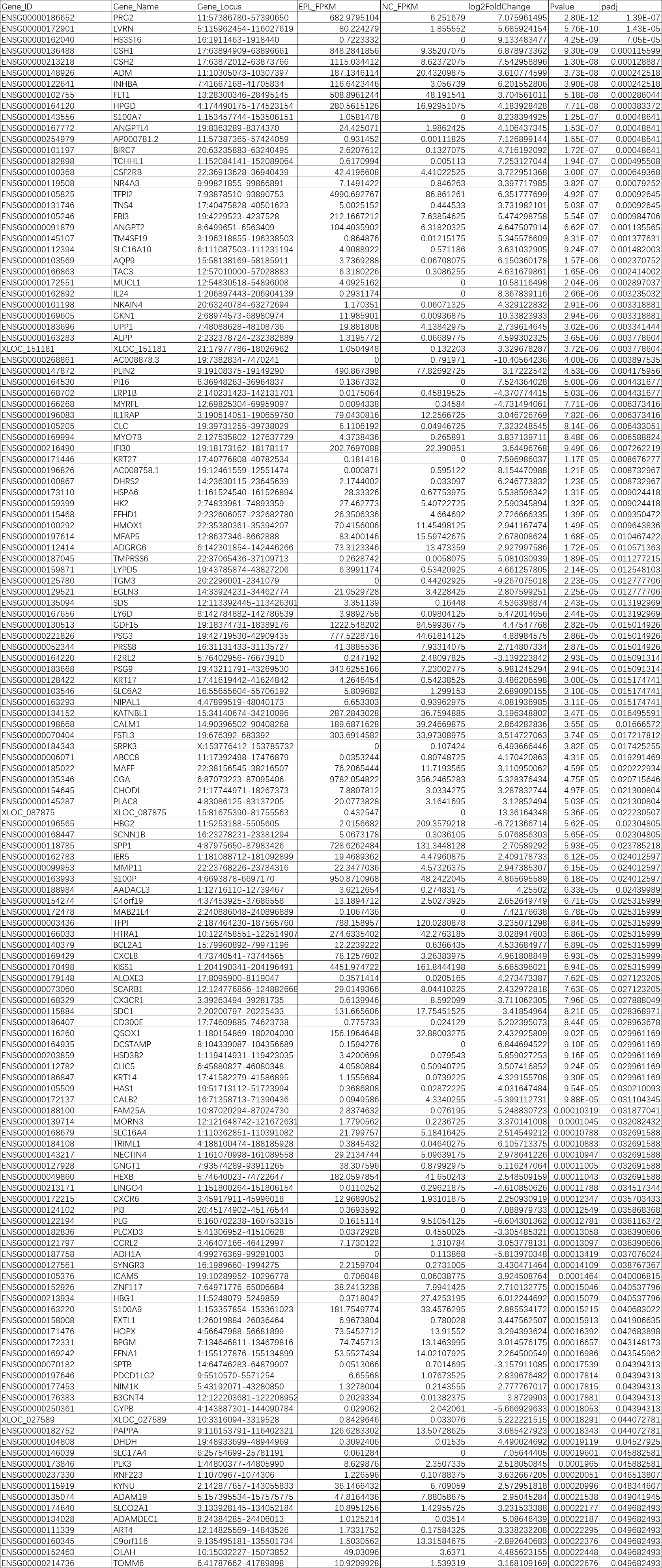

Supplement: Supplementary file 4 — Supplementary file4 (PNG 760 KB) [file 43032_2024_1576_MOESM4_ESM.png]

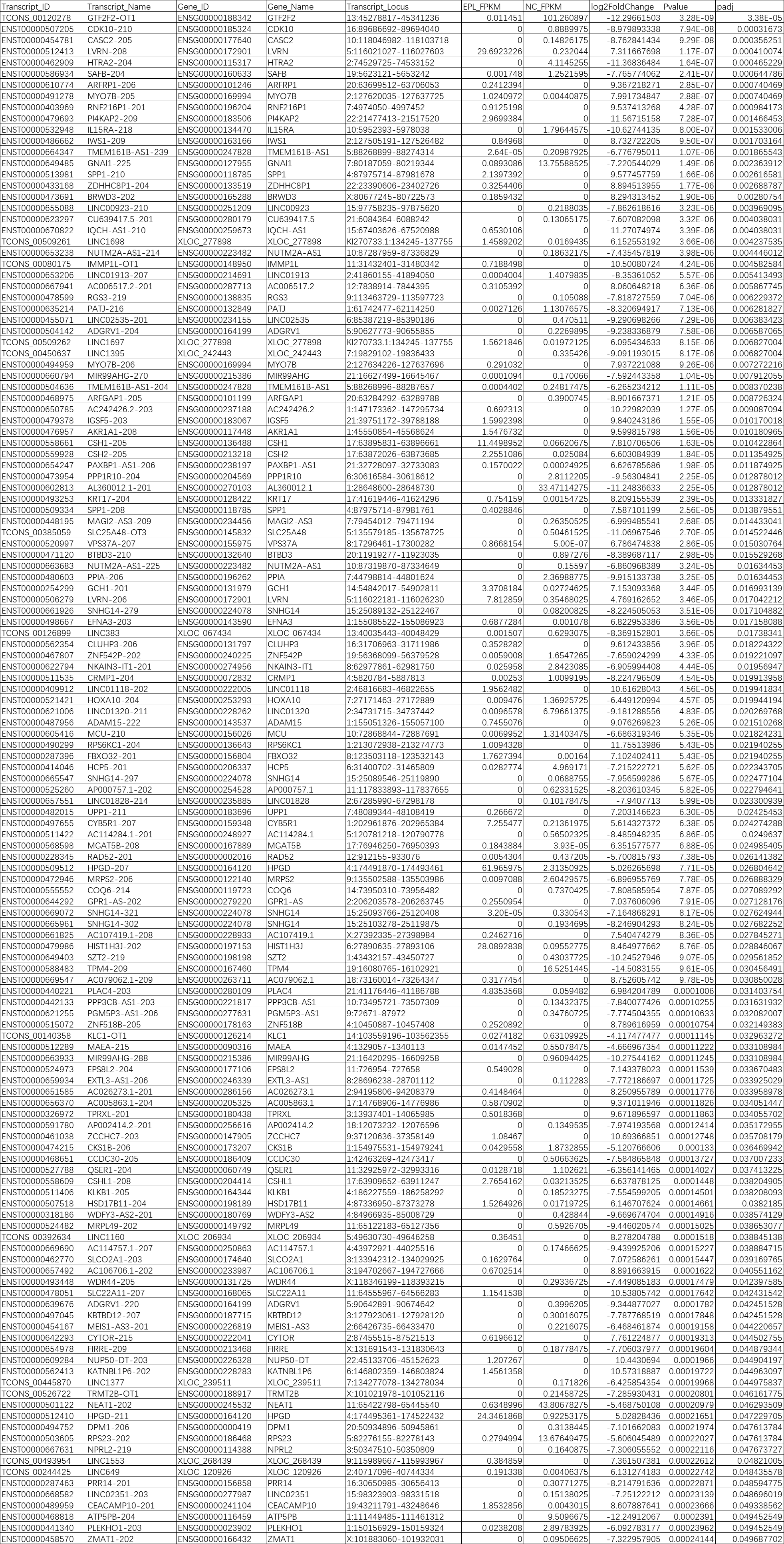

Supplement: Supplementary file 5 — Supplementary file5 (PNG 879 KB) [file 43032_2024_1576_MOESM5_ESM.png]
